# Psoas Cross-Sectional Measurements Using Manual CT Segmentation before and after Endovascular Aortic Repair (EVAR)

**DOI:** 10.3390/jcm11144023

**Published:** 2022-07-12

**Authors:** Caterina Beatrice Monti, Paolo Righini, Maria Chiara Bonanno, Davide Capra, Daniela Mazzaccaro, Matteo Giannetta, Gabriele Maria Nicolino, Giovanni Nano, Francesco Sardanelli, Massimiliano M. Marrocco-Trischitta, Francesco Secchi

**Affiliations:** 1 Department of Biomedical Sciences for Health, Università degli Studi di Milano, 20100 Milan, Italy; caterina.monti@unimi.it (C.B.M.); mariachiara.bonanno@unimi.it (M.C.B.); davide.capra@unimi.it (D.C.); giovanni.nano@unimi.it (G.N.); francesco.sardanelli@grupposandonato.it (F.S.); 2 Unit of Vascular Surgery, IRCCS Policlinico San Donato, 20097 San Donato Milanese, Italy; paolo.righini@grupposandonato.it (P.R.); daniela.mazzaccaro@grupposandonato.it (D.M.); matteo.giannetta@unimi.it (M.G.); 3 Radiology and Diagnostic Imaging Unit, Clinica San Carlo, Paderno Dugnano, 20100 Milan, Italy; gnicolino.md@gmail.com; 4 Unit of Radiology, IRCCS Policlinico San Donato, 20097 San Donato Milanese, Italy; 5 Clinical Research Unit, Cardiovascular Department, IRCCS Policlinico San Donato, 20097 San Donato Milanese, Italy

**Keywords:** sarcopenia, EVAR, psoas muscle, computed tomography

## Abstract

Sarcopenia has been associated with an increased incidence of adverse outcomes, including higher mortality, after endovascular aortic repair (EVAR). We aim to use computed tomography (CT) to quantify changes in total psoas muscles area (PMA) and psoas muscle density (PMD) after EVAR, and to evaluate the reproducibility of both measurements. PMA and PMD were assessed via manual segmentation of the psoas muscle on pre- and post-operative CT scans belonging to consecutive patients who underwent EVAR. Wilcoxon test was used to compare PMA and PMD before and after EVAR, and inter- and intra-reader agreements of both methods were evaluated through Bland–Altman analysis. A total of 50 patients, 42 of them males (84%), were included in the study. PMA changes from 1243 mm^2^ (1006–1445 mm^2^) to 1102 mm^2^ (IQR 937–1331 mm^2^), after EVAR (*p* < 0.001). PMD did not vary between pre-EVAR (33 HU, IQR 26.5–38.7 HU) and post-EVAR (32 HU, IQR 26–37 HU, *p* = 0.630). At inter-reader Bland–Altman analysis, PMA showed a bias of 64.0 mm^2^ and a coefficient of repeatability (CoR) of 359.2 mm^2^, whereas PMD showed a bias of −2.43 HU and a CoR of 6.19 HU. At intra-reader Bland–Altman analysis, PMA showed a bias of −81.1 mm^2^ and a CoR of 394.6 mm^2^, whereas PMD showed a bias of 1.41 HU and a CoR of 6.36 HU. In conclusion, PMA decreases after EVAR. A good intra and inter-reader reproducibility was observed for both PMA and PMD. We thus propose to use PMA during the follow-up of patients who underwent EVAR to monitor muscle depletion after surgery.

## 1. Introduction

Sarcopenia is a progressive and generalized disorder characterized by accelerated loss of skeletal muscle mass, strength and functionality [1]. It was found to be associated with an increase in morbidity and mortality in various conditions such as cancer, and orthopedic and abdominal surgery [2,3,4]. A great range of techniques can be used to assess muscle mass, with no established consensus on a reference standard so far [5].

Measuring psoas muscle area (PMA) on a single cross-sectional abdominal image was found to provide an objective esteem of lean muscle mass [6], while low psoas muscle density (PMD) measured on computed tomography (CT) in Hounsfield units (HU) may be a marker of lipid or fluid infiltration in skeletal muscles, thus indicating the presence of myosteatosis and edema [7,8]. A fatty infiltration of psoas muscle could be related to the presence of endoprothesis.

A pre-operative low PMA, as a surrogate of sarcopenia, has been investigated as a predictor of adverse outcome of patients undergoing endovascular aortic repair (EVAR) of abdominal aortic aneurysm (AAA) in various prior clinical studies [9,10,11,12,13,14,15], whereas PMD has been employed less often for the same purpose [16]. Lindström et al. pointed out the importance of observing also post-operative muscle depletion when considering the prognostic relevance of sarcopenia, as EVAR patients are at high risk of reintervention [17].

Therefore, since CT is routinely performed pre-operatively for surgical planning of EVAR of AAA and post-operatively for follow-up, PMA and PMD may be easily used as biomarkers of sarcopenia in this setting [16,18].

In this study, we use manual CT segmentation to measure PMA and PMD before and after EVAR, with the purpose of quantifying the changes of such parameters in the post-operative setting. We also aim to ascertain the inter- and intra-reader reproducibility of both measurements, to select the biomarker with the best potential for the follow-up of such patients.

## 2. Materials and Methods

### 2.1. Patients

The local ethics committee (Ethics Committee of IRCCS Ospedale San Raffaele) approved this study (protocol code “CardioRetro”, number 122/int/2017; approved on 14 September 2017, and amended on 10 February 2021). Informed consent was waived due to the retrospective nature of the study.

We retrospectively selected consecutive patients who were treated for AAA by EVAR at our Institution from September 2010 to May 2019 and underwent both a preoperative CT scan in the year before EVAR and a follow-up CT scan at least 2 months after EVAR.

### 2.2. Image Acquisition and Post-Processing

Patients were studied using 1 of 2 different multidetector scanners, one 64-slice unit and one 16-slice unit. For the 64-slice CT unit (Somatom Definition, Siemens Healthineers, Erlangen, Germany), the following technical parameters were used: tube voltage 120 kVp; tube current from 100 to 200 mAs, depending on automatic exposure control system (CARE Dose 4D, Siemens); rotation time 0.5 s; pitch 1; slice thickness 5 mm; kernel reconstruction technique B30f medium smooth. For the 16-slice CT unit (Emotion 16, Siemens), the following technical parameters were used: tube voltage130 kVp; tube current from 100 to 200 mAs, depending on automatic exposure control system (CARE Dose 4D, Siemens), rotation time 0.5 s; pitch 1; slice thickness 5 mm; kernel reconstruction technique B30f medium smooth. Abdominal aortic non-enhanced and contrast-enhanced CT were acquired.

All CT images were reviewed using a dedicated medical imaging workstation (Carestream Vue PACS viewer version 12.1.0.0365 Rochester, NY, USA). Non-enhanced images were preferred for density and area measurements to avoid the changes in muscle attenuation caused by intravenous contrast material [19].

**Figure 1:**
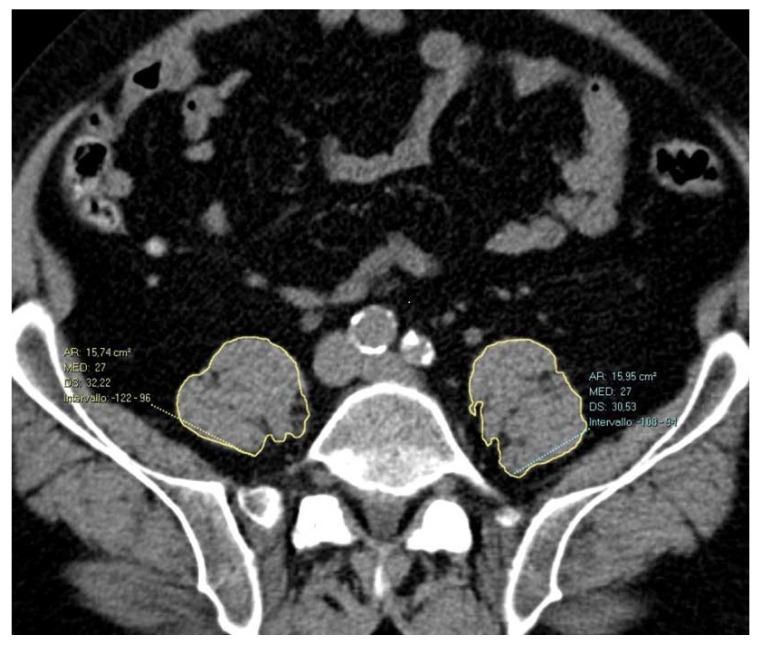
Axial non-enhanced CT scan showing polygonal manual segmentation (yellow outline of bilateral psoas muscles). The right psoas muscle area is 1574 mm^2^, and the left psoas muscle area is 1595 cm^2^. The PMD is 27 HU on both sides.

The axial series of non-enhanced images of both pre- and post-operative CT was hence opened as a multiplanar reconstruction view to identify the desired slice at the inferior endplate of the 4th lumbar vertebra (L4) [20]. In the axial plane, a region of interest (ROI) was traced manually, outlining the area of each psoas muscle, as shown in Figure 1, and the combined total area enclosed was recorded as PMA as well as the average density as PMD. To evaluate inter-reader agreement, on pre-operative images, PMA and PMD were blindly measured by two radiology residents with 1 and 4 years of experience (R1, R2) trained by a board-certificated radiologist in CT imaging. To calculate intra-reader reproducibility, one of the two residents repeated the measurements two weeks later.

Finally, the total number of lumbar arteries was calculated before and after EVAR.

### 2.3. Statistical Analysis

Categorical variables were summarized by percentages and continuous scale variables were expressed as median ± interquartile range (IQR).

Each variable of interest was tested for normality using the Shapiro–Wilk test.

Wilcoxon test was used to compare measurements before and after EVAR; using only the right side area to reduce the variability.

Inter- and intra-reader reproducibility was evaluated using Bland–Altman method. For this purpose, given the lack of significant differences between psoas areas on the two sides [21], the combined total area of both sides (tPMA) was used to ascertain the exact reproducibility of measurements.

Statistical analysis was performed with SPSS v20 (IBM SPSS Inc., Chicago, IL, USA). *p*-values smaller than 0.05 were considered indicative of statistical significance.

## 3. Results

### 3.1. Demographics and Clinical Data

A total of 50 patients were included in the study. No patients in our population underwent an iliac artery embolization. Demographic and clinical data for the study population are summarized in Table 1.

### 3.2. Changes in CT Parameters before and after Surgery

**Figure 2:**
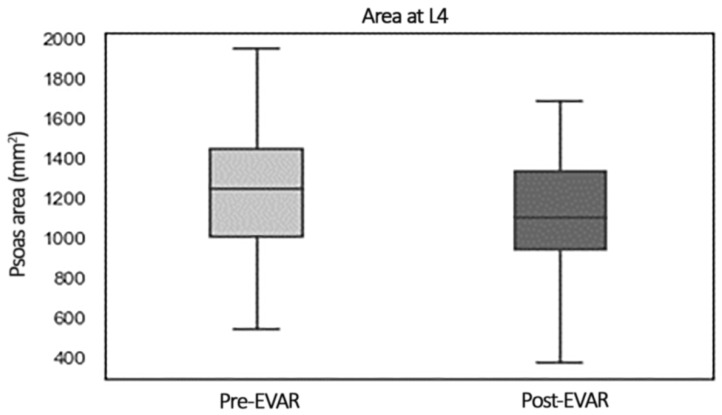
Box plot showing comparison of PMA before and after EVAR.

PMA after EVAR decreased significantly (*p* < 0.001) from 1243 mm^2^ (1006–1445 mm^2^) to 1102 mm^2^ (IQR 937–1331 mm^2^), as shown in the plot box in Figure 2. PMD did not vary from a pre-EVAR of 33 HU (26.5–38.7 HU) to a post-EVAR of 32 HU (26–37 HU, *p* = 0.630).

A total of lumbar arteries before EVAR was 6 (6–8) and after EVAR 4.5 (1–6) with a significant difference (*p* < 0.001).

### 3.3. Inter- and Intra-Reader Reproducibility

R1 obtained a PMA value of 2535.5 mm^2^ (2067.2–2818 mm^2^) and a PMD of 34.5 HU (26.6–37.8 HU) for the first reading, and a PMA of 2535 mm^2^ (2034.5–2831.5 mm^2^) and a PMD of 31.5 HU (25.7–38 HU) for the second reading. R2 obtained a PMA of 2631.5 mm^2^ (2050.2–2877.7 mm^2^) and a PMD of 32 HU (25.1–37.5 HU).

Intra-reader reproducibility of PMA showed a bias of −81.1 mm^2^ and CoR of 394.6 mm^2^; reproducibility of PMD showed a bias of 1.41 HU and Cor of 6.36 HU.

**Figure 3:**
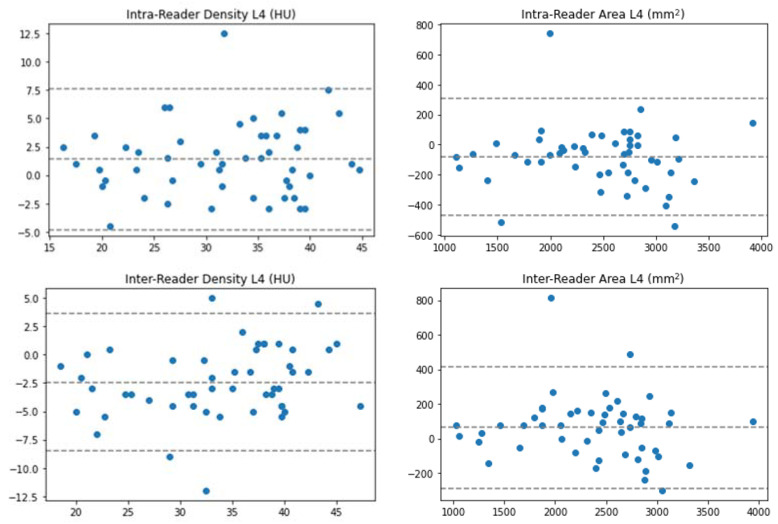
Bland–Altman plots for intra- and inter-reader reproducibility.

Inter-reader reproducibility of PMA showed a bias of 64.0 mm^2^ and CoR of 359.2 mm^2^; reproducibility of PMD showed a bias of −2.43 HU and CoR of 6.19 HU. Bland–Altman plots are shown in Figure 3.

## 4. Discussion

In this study, we observed a significant decrease in PMA after EVAR, compared to pre-operative imaging, while no significant difference was found in PMD values, although both measurements showed a good reproducibility.

Sarcopenia is a progressive and generalized skeletal muscle disorder, whose diagnostic pathway includes measurements of muscle mass, muscle strength and physical performance [1]. While this condition is well known to the aging literature, recent studies have emphasized its relevance in various clinical settings, such as cancer and surgery [2,4]. As a result, the term sarcopenia is currently used as a synonym for low muscle mass and myosteatosis is often considered as an aspect of the disorder, rather than a distinct entity [22]. Despite the inconsistency of such terminology, CT assessment of muscle area and attenuation has become a useful tool for evaluating skeletal muscle status in a growing number of research studies [23].

AAA is a relatively common and potentially life-threatening disorder. Despite a significant reduction in mortality after the introduction of EVAR, the 5-year survival after successful repair of AAA remains below 70% [23]. For this reason new mortality risk scoring schemes have been developed to stratify patients under consideration for planned open repair or EVAR [24].

Since PMA has started to be used as a surrogate of sarcopenia, many studies have focused on the assessment of low values of PMA before the intervention of EVAR in order to evaluate its prognostic role and find a tool for risk stratification of surgical candidates [9,10,11,12,13,14,15]. Pre-operative low values of PMA have been found to predict poor outcomes, such as reduced long-term survival and longer hospital stay [11,14]. By comparison, psoas muscle attenuation has instead been used significantly less than PMA to predict the outcome of EVAR [16].

However, less attention has been paid to the decrease in PMA after EVAR and its possible implication in time of recovery and residual functional impairment. While post-operative dynamic change in skeletal muscle mass has been investigated after major abdominal surgeries [25,26], to the best of our knowledge, only two studies have focused on relative post-operative decrease of PMA after EVAR.

Lindström et al. [17] for the first time urged the need of studying muscle mass changes also after EVAR as, since these patients are at high risk of reintervention, they thought necessary to stratify those who would truly benefit from additional procedures. For this purpose, they used PMA to describe the advance of sarcopenia after EVAR and found a negative correlation between muscle depletion and long-term survival.

Ouchi et al. [27] observed a significant decrease in PMA of patients who had undergone EVAR compared to a control group of patients who were treated with thoracic EVAR. Psoas muscles receive blood from different arteries, such as lumbar, iliolumbar, deep circumflex iliac, external iliac and femoral. As lumbar arteries are branches of the abdominal aorta at the level of the L1 to L4 vertebrae the authors suggested that the decrease in PMA may be attributed to ischemic injury caused by the obstruction of blood across these vessels due to the insertion of an aortic stent graft [27]. A confirmation of this observation is provided by our data. We found a significant reduction in the number of lumbar arteries after EVAR. A larger study is needed to evaluate the role of sarcopenia and that of lumbar arteries occlusion in the reduction of PMA.

The present study observed a significant decrease in PMA after EVAR, compared to pre-operative CT. Our results are consistent with those obtained by Lindström et al. [17], who also observed that the most significant loss of PMA after surgery occurs during the first year post-operation, with a mean proportional difference of −26.8% [17]. Although we encountered a smaller relative reduction (representing 6.86% of the pre-EVAR area), probably because of the large variability in ranges of interval between the intervention and the first follow-up available.

In addition, we have demonstrated that measuring PMA using tools available within a clinic setting has acceptable reproducibility between observers. Therefore, we propose the use of PMA during the follow-up of patients who underwent EVAR to monitor muscle depletion after surgery. Early recognition of this condition is essential to time nutritional intervention and physical exercise, to postpone functional decline and therefore, as psoas muscle plays a fundamental role in basic activities such as standing and walking, improve quality of life [28].

In this paper, we also investigated changes in PMD after EVAR. However, PMD did not show a statistically significant change after the intervention, therefore we do not advise the use of PMD for follow-up, even if both measures showed a high reproducibility.

Our study has some limitations, firstly due to its nature as a single-center retrospective observational study. Second, the overall number of patients was low, although we were still able to obtain significant results. Thirdly, there was a broad variability in ranges of interval between operation and post-operative examination, therefore the muscle mass of patients may be deteriorated for reasons other than the intervention itself.

This is a preliminary study, and in the future further research should aim to narrow the interval range between EVAR and the follow-up CT scans and investigate the impact of post-operative muscle depletion on long-term outcome.

Moreover, we hope that our results might encourage future studies to evaluate the correlation between postoperative decrease in PMA and functional muscle status, with appropriate tests such as gait speed and exhaustion, as well as consider the subjective symptoms of patients and the impact on their daily life.

## 5. Conclusions

PMA shows a significant reduction after EVAR and given its high inter- and intra-reader reproducibility it could represent a viable biomarker for the post-operative follow-up of these patients.

In clinical use, PMA may allow a prompt detection of muscular decline in the early post-surgical setting and implement targeted intervention by mean of physical rehabilitation.

## Figures and Tables

**Table 1:** Demographics and clinical data.

Variable	N (%) or Median (IQR)
Demographics	
Male	42 (84)
Age (years)	72 (66–78)
Follow up time (months)	12 (8–19)
Comorbidities and risk factors	
Hypertension	40 (80)
Diabetes	7 (14)
CAD	14 (28)
Kidney failure	6 (12)
COPD	15 (30)
Smokers	23 (46)
Anticoagulant treatment	2 (4)

## Data Availability

Data is available upon reasonable request to the corresponding author.
